# Familial Hemophagocytic Lymphohistiocytosis May Present during Adulthood: Clinical and Genetic Features of a Small Series

**DOI:** 10.1371/journal.pone.0044649

**Published:** 2012-09-07

**Authors:** Elena Sieni, Valentina Cetica, Andrea Piccin, Filippo Gherlinzoni, Ferdinando Carlo Sasso, Marco Rabusin, Luciano Attard, Alberto Bosi, Daniela Pende, Lorenzo Moretta, Maurizio Aricò

**Affiliations:** 1 Department Pediatric Hematology Oncology, Azienda Ospedaliero Universitaria A. Meyer Children Hospital, Firenze, Italy; 2 Hematology Department and BMT Unit, San Maurizio Regional Hospital, Bolzano, South Tyrol, Italy; 3 Divisione di Ematologia, Department of Medicina Specialistica, U.L.S.S. 9 -Ospedale "S. Maria di Ca' Foncello", Treviso, Italy; 4 Department of Internal and Experimental Medicine, Second University of Naples, Naples, Italy; 5 I.R.C.C.S. Burlo Garofolo, UO Pediatric Hemato-Oncology, Trieste, Italy; 6 Infectious Diseases, Azienda Ospedaliero Universitaria S.Orsola-Malpighi, Bologna, Italy; 7 Hematology Department, Azienda Ospedaliero Universitaria Careggi, Firenze, Italy; 8 IRCCS A.O.U. San Martino-IST, Genova, Italy; 9 IRCCS Giannina Gaslini, Genova, Italy; Instituto de Ciencia de Materiales de Madrid - Instituto de Biomedicina de Valencia, Spain

## Abstract

Familial Hemophagocytic lymphohistiocytosis (FHL) is a rare immune deficiency with defective cytotoxic function. The age at onset is usually young and the natural course is rapidly fatal if untreated. A later onset of the disease has been sporadically reported even in adolescents and adults. We report the results of our retrospective data collection of all cases diagnosed with FHL at an age of 18 years or older and enrolled in the Italian Registry of HLH. All cases were diagnosed with FHL based on evidence of genetic defect in one FHL-related gene. A total of 11 patients were diagnosed with FHL. They were 9 males and 2 females, from 10 unrelated families; their age ranged between 18 and 43 years (median, 23 years). Family history was unremarkable in eight families at the time of the diagnosis. Their genetic diagnoses are: FHL2 (n = 6), FHL3 (n = 2), FHL5 (n = 1), XLP1 (n = 2). Clinical, molecular and functional data are described. These data confirm that FHL may present beyond the pediatric age and up to the fifth decade. FHL2 due to perforin defect is the most frequently reported subtype. Adult specialists should consider FHL in the differential diagnosis of patients with cytopenia and liver or central nervous system disorders, especially when a lymphoproliferative disease is suspected but eventually not confirmed. FHL may turn to be fatal within a short time course even in adults. This risk, together with the continuous improvement in the transplant technique, especially in the area of transplant from matched unrelated donor, resulting in reduced treatment related mortality, might suggest a wider use of SCT in this population. Current diagnostic approach allows prompt identification of patients by flow-cytometry screening, then confirmed by the genetic study, and treatment with chemo-immunotherapy followed by stem cell transplantation.

## Introduction

Familial Hemophagocytic lymphohistiocytosis (FHL, OMIM 267700) is a genetically heterogeneous disorder characterized by a hyper-inflammatory syndrome with fever, hepatosplenomegaly, cytopenia and sometimes central nervous system involvement. Bone marrow aspiration is usually performed early during the diagnostic work-up, enabling the identification of haemophagocytosis by activated macrophages [1]. In most cases the natural course of FHL is rapidly fatal within a few weeks, unless appropriate treatment, including corticosteroids, cyclosporine, etoposide, anti-thymocyteglobulin, can obtain transient disease control. So far, only patients who underwent hematopoietic stem cell transplantation have been cured [1]–[5].

Diagnosing FHL has been challenging for clinicians for many years. Initially, the key points for the diagnosis of HLH have been the diagnostic criteria, established by the Histiocyte Society, collecting both clinical and biochemical features, all rapidly available to the attending physician. Recently, the diagnostic criteria have been updated to introduce sCD25 level but, even more important, hyperferritenemia and defective natural killer activity [6]. Better understanding of the pathogenic mechanisms of FHL provided the rationale for developing novel, sensitive and specific diagnostic tools based on flow-cytometry analysis of peripheral blood cells. In particular, evidence of defective expression of intra-cytoplasmic perforin, as well as defective degranulation upon stimulation of the cytotoxic effector cells, provided very potent tools for initial screening of patients with suspected FHL [7], [8].

FHL is associated with an overactive adaptive immune system, probably resulting from the failure of activated CTLs and NK cells to clear APCs and therefore to terminate an immune response [9]–[11]. The uncontrolled expansion and activation of polyclonal CD8+ T cells may lead to macrophage activation, with infiltration of tissues and organs, together with excessive release of inflammatory cytokines which also causes tissue damaging.

Since 1999, several genes have been associated with FHL: *PRF1 (*OMIM *170280), *UNC13-D (*OMIM *608897*), STXBP-2* (OMIM *601717) and *Syntaxin-11* (OMIM *605014), all encoding proteins which play a key role in lymphocyte cytotoxicity [12]–[16]. Additional congenital immune deficiencies may expose the patient to develop a clinical picture overlapping FHL. Patients with Chediak-Higashi syndrome have biallelic mutations of the gene encoding the cytoplasmic protein lysosomal trafficking regulator (LYST;OMIM*606897) and have granulated cells with giant intracytoplasmic lysosomal structures. Griscelli syndrome type 2 (GS2) is characterized by hypopigmentation (owing to defective release of melanosome contents from melanocyte dendrites) and defective CTL and NK cell cytotoxic activity. Biallelic mutations in the gene encoding RAB27a (OMIM*603868) are responsible for GS2 [11], [17]. Finally, several primary immunodeficiency syndromes, including X-linked lymphoproliferative disease (XLP) type I due to hemizygous mutation of *SH2D1A*gene, OMIM *300490), and type II due to mutation of *BIRC4/XIAP* gene (OMIM *300079) are associated with a high risk of developing HLH [18], [19].

The age at diagnosis of FHL is often very young. Since the original report of two affected siblings aged nine weeks by James Farquhar in 1952 [20], FHL has widely been considered as a disease characteristic of the first two years of age. This may mislead the clinician into conclude that a patient with a clinical constellation resembling HLH is simply considered to be “too old” for this diagnosis. Over the years, sporadic reports of older children and adolescents have raised the issue of a possible “threshold” age for diagnosing FHL [21], [22].

Here we report a series of patients with FHL due to a documented genetic defect who developed the disease during adulthood.

## Results

A total of eleven patients were diagnosed with FHL based on the finding of mutations in one FHL-related gene. They included 9 males and 2 females, from ten unrelated families; their age ranged between 18 years and 43 years (median, 23 years). Their main presenting features are summarized in Table 1. Nine families were of Italian origin, and one was from Colombia. Family history was unremarkable in eight families at the time of the diagnosis. Consanguinity was reported in two families.

**Table 1:** Main features of eleven adults with FHL.

UPN	Gender/Age (years)	Diagnosis	Gene/Protein	Genotype	Fever	Splenomegaly	Platelet (count/mm^3^)	Fibrinogen (mg/dl)	Ferritin (ng/ml)	Hemophagocytosis	Functional study	Course and outcome
198	M/18	FHL3	UNC13D/Munc13–4	c.1847A>G p. E616Gc.1847A>G p. E616G	+	+	25	90	NP	+	Absent NK cell cytotoxicity	Response to initial therapy according to HLH94, dead of septicemia at 18.8 years
199	M/18	XLP1	*SH2D1A*/SAP	Hemizygous del Exon1	+	+	19	120	NP	+	Not performed	Early death of progressive disease. Diagnosis confirmed after death
232	M/27	FHL 2	*PRF1*/Perforin	c.272C>T p. A91Vc.1122G>A p.W374X	+	+	25	<100	>10,000	+	Absent perforin expression and NK cell cytotoxicity	Refused allogeneic SCT, dead of progressive disease at 36 years
233	F/22	FHL 2	*PRF1*/Perforin	c.272C>T p. A91Vc.1122G>A p.W374X	+	+	20	<100	>10,000	-	Absent perforin expression and NK cell cytotoxicity	Progressive diseases at 37 years, awaiting for SCT
276	M/22	FHL 2	*PRF1*/Perforin	c.160C>T p. R54Cc.272C>T p. A91V	+	+	25	NP	NP	+	Not performed	Refused SCT, early death of progressive disease
525	M/23	FHL 5	*STXBP2*/Munc18–2	c.416C>G p.P139R*c.1247-1 G>C p.SPLICE error	+	+	19	120	13,640	-	Defective protein, degranulation and NK cell cytotoxicity	Response to initial therapy according to HLH2004, cured after SCT
557	M/39	FHL 2	*PRF1*/Perforin	c.452A>T p.H151L*c.452A>T p.H151L*	+	+	30	<100	NP	-	Not Performers	Dead of progressive disease during initial therapy according to HLH2004
577	F/28	FHL 2	*PRF1*/Perforin	c.695G>A p.R232Hc.673C>T p. R225W	+	+	9	69	295	+	Not performed	Early death of progressive disease
667	M/43	FHL 2	*PRF1*/Perforin	c.695G>A p.R232Hc.1099T>C p.Y367H*c.272C>T p. A91V	+	+	35	440	6,113	+	Absent perforin expression	Initial response to therapy according to HLH2004, dead of early reactivation before SCT
705	M/19	XLP1	*SH2D1A*/SAP	c.3G>C p.M1I	+	+	110	<100	5,800	-	Normal perforin expression and degranulation	Response to initial therapy according to HLH-94, waiting for SCT
715	M/25	FHL 3	UNC13D/Munc13–4	c.2346_2349delGGAGp. R782SFsX11c.2459T>G p.L850R	+	+	24	93	33,660	-	Defective degranulation	Rensponse to initial therapy according to HLH-2004, waiting for SCT

* Novel mutation;

Patient 198 developed the disease at 18 years with a typical picture. Specific therapy was rapidly followed by disease control, but unfortunately the child died of complication within a few months. Patient 199 had been diagnosed with fulminant mononucleosis, and the diagnosis of HLH was made when he was at a terminal stage. XLP1 was then diagnosed retrospectively.

Patient 232 and 233 belong to the same family, and preliminary data had been reported soon after the diagnosis [22]. Patients 232 developed, at 22 years, isolated, asymptomatic hypertransaminasemia, followed, a few months later, by left arm weakness lasting one month; then, at 27 years, again fever, lymphadenopathy, pancytopenia and neurologic symptoms (hemiplegia and somnolence). Multifocal brain demyelination lead to a diagnosis of neurosarcoidosis, treated with high doses i.v. glucocorticoid with good clinical response. Oral prednisone was progressively tapered but a minimal dose of 40 mg daily was required to control fever and asthenia, while pancytopenia persisted, with recurrent respiratory and urinary infections. We first saw him two years later with fever, dyspnea, herpes simplex lesions in nasal and perioral skin, marked hepatosplenomegaly, and severe Cushingoid features; pancytopenia, hypofibrinogenemia, hypertriglyceridemia, high levels of transaminases, cholestatic indexes, and ferritin, with hemophagocytosis were observed. He refused allergenicstem cell transplantation (SCT) and remained clinically stable for 10 years until eventually developing full-blown FHL with severe CNS involvement, which turned to be fatal. Family history at the time of the diagnosis had revealed that his sister (patient 233), had been diagnosed with non-Hodgkin lymphoma and treated with chemotherapy followed by autologous SCT. Diagnostic work-up confirmed FHL2 redirecting interpretation of the initial episode (fever, weight loss, weakness, hepatosplenomegaly, pancytopenia, hypertriglyceridemia, hypofibrinogenemia, hyperferritinemia, with polyclonal T-cell lymphoproliferation). She remained asymptomatic for over 10 years but eventually developed full-blown FHL at the age of 37 years. At the time of writing she is being treated with allogeneic SCT at another centre.

Patient 276 developed full-blown HLH at 22 years but refused treatment with a rapidly fatal course.

The medical history of patient 525 revealed tonsillectomy at the age of 5 years and two other similar 'infectious mononucleosis like' bouts at the age of 7 and 22 years, which resolved spontaneously. At the age of 23 years he had another similar episode, which gradually recovered within 10 days. However, a month later he was readmitted with a similar picture, ascites and pleural effusion. HLH was suspected and confirmed by the functional and genetic study. Following chemo-immunotherapy according to HLH-2004 protocol, he achieved disease control and was then successfully treated with allogeneic SCT.

Patient 557 was diagnosed with full-blown FHL occurring as his first relevant health problem at the age of 39 year. FHL2 was diagnosed based on absent perforin expression and biallelic mutations, compatible with parental consanguinity. Unfortunately he died during initial therapy due to progressive disease. Soon thereafter, during the genetic study of the family, his 17-month-old daughter was found to be compound heterozygous due to a maternal nonsense *PRF* mutation and developed full-blown FHL. One elder sister of patient 557 had died at the age of 16 year of progressive encephalopathy with cytopenia, and genetic confirmation of her diagnosis of FHL awaits availability of diagnostic tissue.

Patients 577 and 667 developed full-blown, rapidly progressive FHL as their first major health problem at 28 and 43 years, respectively.

Patient 705 developed, at the age of 19 years, a typical HLH, diagnosed as so in one of our pediatric centers. Patient 715 was admitted to an Infectious disease academic unit where HLH was suspected and then confirmed by the centralized diagnosis.

## Discussion

Most of the available information on the clinical spectrum and natural course of FHL derives from reports of children diagnosed by a few referral centres in Europe, North America or Japan [1]–[2]. This lead to the common belief that FHL is restricted to children or infants, and does not pertain to adult patients. Although the vast majority of patients did indeed develop the disease during the first few years of life, the concept that later onset of HLH is possible has been repeatedly brought to the attention of adult haematologists [21], [22], [26]. Nevertheless, adult patients are at most considered as potentially affected by the so-called “secondary” form of HLH, also defined in the past as “virus (or infection) associated hemophagocytic syndrome” (VAHS or IAHS) [27] rather than by genetic FHL. This may have several implications, the most important being reluctance to apply a complete, standard therapy for FHL, which at present consists of chemo-immunotherapy with dexamethasone and etoposide, then followed by cyclosporine, as defined by the HLH94 study [2], [28]; its primary aim is to achieve disease control and then forward the patient to allogeneic SCT, the only currently available treatment with potential for cure [2], [4]–[5]. Genotype-phenotype studies of FHL have established a close correlation between biallelic disruptive mutations and early age at the onset of FHL [29], [30]. In the presence of hypomorphic genetic defects, residual protein allows some level of residual NK- and T-cell function, which may become sufficient to cope with infectious agents, the usual triggers of FHL, for many years. We report on six patients with FHL2 due to compound heterozygosity, in whom at least one allele carried a missense mutation. In four of these six, it was the PRF1 A91V single nucleotide change. Although its pathogenic role has been widely debated, due to its 2–5% frequency at the heterozygous state in control population, its pathogenic role on perforin protein has been clearly documented [23], [29].

In a recent similar report, Zhang et al. described 10 adult patients with FHL due to biallelic mutations; of them 7 had FHL2, and A91V was found in 5 of the 14 mutated alleles [31].

Based on the pediatric experience, diagnostic criteria have been developed. This set of eight clinical and laboratory items, which proved to be sufficiently sensitive and specific, might be updated using a more practical clinical approach. Patients with fever, splenomegaly, thrombocytopenia and high levels of ferritin, in whom bone marrow aspirate does not document leukemia, should be rapidly screened by flow-cytometry for the expression of intracytoplasmic perforin and the ability to degranulate– as measured by surface expression of CD107a upon appropriate stimulation – of the peripheral blood lymphocytes [8]. This procedure, widely accessible to haematology or immunology laboratories, can predict the presence of mutations in the *PRF1* or in the *UNC13D, STX11, STXBP2* genes, respectively [32]. This approach may be expected to cover most of the cases of FHL, thus providing a robust support to the diagnosis and justification for high-risk treatment in life-threatening conditions, including indication to SCT.

The question about indication to SCT for patients with milder clinical picture or even in a presymptomatic phase remains open. Increasing body of evidence suggests that full-blown FHL may turn to be fatal within a short time following the diagnosis, even in adults with hypomorphic mutations. This risk, together with the continuous improvement in the transplant technique, especially in the area of transplant from matched unrelated donor, resulting in reduced treatment related mortality, might suggest a wider use of SCT in this population.

## Methods

### Setting

Starting from 1984 a Registry for HLH was established [1]. The pediatric community in the country was offered the opportunity to centralize patient information and biologic samples, in order to confirm the clinical diagnosis and to perform centralized immunological and genetic studies, since the time these last became available.

Hemophagocytic Lymphohistiocytosis was defined by the diagnostic criteria established by the Histiocyte Society [6].

### Immunological Analyses

Peripheral blood mononuclear cells (PBMC) from FHL patients and healthy donors were isolated by Ficoll gradient centrifugation. NK cells were also purified using the RosetteSep method (StemCell Technologies, Vancouver, British Columbia, Canada) following manufacturer’s instructions. NK cells were cultured on irradiated feeder cells in the presence of 2 µg/mL phytohemagglutinin (Sigma-Aldrich, Irvine, UK) and 100 U/mL rIL-2 (Proleukin, Chiron Corp., Emeryville, USA) to obtain high numbers of polyclonal activated NK cell populations.

Perforin expression on NK cells (CD3^−^CD56^+^ cells of PBL or purified activated NK populations) was detected by intracellular staining (after fixation and permeabilization) with ΔG9 mAb and cytofluorimetric analysis, as previously reported [23].

To analyse the cytolytic activity in 4-h ^51^Cr-release assays, PBMC were tested against K562, while activated NK cells were tested against the HLA-class I^-^ B-EBV cell line 721.221, demonstrated to be suitable effector/target combinations to reveal cytolytic defect of FHL patients [8]. E:T ratios ranging from 100∶1 to 1∶1 were used for PBMC as effector cells, while from 8∶1 to 0.5∶1 for activated NK. Lytic units (LU) at 30% lysis were calculated. Resting and activated NK cells were also tested in degranulation assay quantifying cell surface CD107a expression upon co-culture with K562, as previously described [8]. Briefly, anti-CD107a-PE mAb was added during the co-culture for 3 hours at 37°C in 5% CO2. Thereafter, the cells were stained with anti-CD56-APC and anti-CD3-PerCP mAb and analysed by flow cytometry (FACSCalibur, Becton Dickinson). All reagents were from BD Biosciences (Oxford, UK). Surface expression of CD107a was assessed in the CD3^-^CD56^+^ cells. Results were evaluated as ΔCD107a (i.e. % CD107a^+^ cells of stimulated - % CD107a^+^ cells of unstimulated sample) and defined defective when lower than the 10^th^ percentile of healthy controls.

Expression of Munc13-4 and Munc18-2 proteins was analyzed by western blot using cell extracts and specific polyclonal antibodies, as previously described [24], [25].

### Mutation Analysis

Genomic DNA was isolated from peripheral blood samples using BioRobot® EZ1 Workstation (Qiagen, Jesi Italy) or Qiaamp DNA Mini Kit (Qiagen, Jesi Italy), then amplified and directly sequenced, in both directions, with the BigDye® Terminator Cycle SequencingReady Reaction Kit (Applied Biosystems, Foster City, CA, USA). Amplification reactions were performed with 60 ng of DNA, 10 ng of each primer, 200 µM dNTPs, 1x PCR reaction buffer, and 2.5 U Taq polymerase in a final volume of 25µl. Sequences obtained using an ABI Prism® 3130XL Sequence DetectionSystem (Applied Biosystems) were analysed and compared with the reported gene structure using the dedicated software SeqScape® (Applied Biosystems). All mutations were confirmed in the parents.

*PRF1, UNC13D, STXBP2 and SH2D1A* genes have been analyzed. All patients underwent direct sequencing of PCR products spanning the coding exons and exon–intron boundaries of the four selected genes. The sequences obtained were compared with the reported gene structures (PRF1 gene number: 190339, NCBI; *UNC13D* gene number: 201294, NCBI; *STXBP2* gene number: 6813, NCBI; *SH2D1A* gene number: 4068, NCBI).

Data were collected and stored in a specific Microsoft Access data-base.

### Ethics Statement

Written informed consent for the data collection including enrollment in the Registry, the functional study, and the genetic study was obtained by the attending physician for all patients as well as from all healthy donors. The study was approved by the IRB of the AOU Meyer, Florence. All clinical investigations were conducted according to the principles expressed in the Declaration of Helsinki.
